# Spatial Disparity of Visitors Changes during Particulate Matter Warning Using Big Data Focused on Seoul, Korea

**DOI:** 10.3390/ijerph19116478

**Published:** 2022-05-26

**Authors:** Sang-Hyeok Lee, Jung Eun Kang

**Affiliations:** Department of Urban Planning and Engineering, Pusan National University, Busan 46241, Korea; ubscott@pusan.ac.kr

**Keywords:** particulate matter, visiting population, spatial disparity, hotspot, Seoul

## Abstract

This study examined the changes in the number of visitors to regions during periods of high particulate matter (PM) concentrations in Seoul and analyzed the regional differences of these changes. Further, it examined the spatial characteristics that affect these regional differences. This study mapped the regional differences by conducting a spatial cluster analysis using GIS and examined factors affecting the regional differences using logistic regression analysis. The visiting population data used in this study were obtained from the Big Data on the de facto population measured every hour at mobile base stations, and all analyses were conducted in terms of weekdays and weekends. The results indicated that the visiting population decreases significantly on weekdays when there are high PM concentrations; however, visits increase on weekends, even during periods of high PM concentrations. Moreover, there was a huge regional gap in visiting population changes. Regions with more commercial use, higher bus accessibility, and better pedestrian environment (pedestrian paths, Walk Score) were more likely to be hotspots, whereas regions with high residential and industrial use were more likely to be cold spots. These results can be used as the basic data for PM policies based on regional characteristics.

## 1. Introduction

Cities are often sites of massive human movement and agglomeration; thus, various forms of environmental problems that deteriorate the living environment for urban residents have received a great amount of attention. Koreans consider particulate matter (PM) as the most serious environmental problem affecting them, and anxiety over PM is much higher than that over radioactivity, harmful chemical substances, or climate change [1]. This high anxiety is fueled by frequent advisories and warnings on PM, heightened media attention, and an increased interest in health among individuals.

Studies on the impact of PM have been conducted in various fields such as environment, health, and urban planning. Studies focused on PM’s effect on individuals’ health have found that along with increasing the prevalence of respiratory and cardiovascular diseases, PM also deteriorates individual health and activity, thereby reducing the activity of urban residents through behavioral changes and decreased willingness to act due to its emotional and mental impact [2,3,4,5,6]. Most studies have analyzed PM’s impact on individual health or activity by utilizing survey data; few studies have collectively examined individuals and spatially analyzed the effect of PM using objective data.

The reason for the difficulty in such research is the absence of data and methodology to measure the activities of urban residents that can be used for spatial analysis. However, with the recent development of communication, sensor, and data-processing technology, it has become possible to collect population information by time and purpose through mobile base stations. Such population information provides an opportunity to conduct various studies on the space–time distribution and movement of the population; the high-resolution floating population Big Data have begun to be utilized in research on the dynamics of urban residents [7,8,9]. Spatial exploration using high-resolution population information on the effects of PM on the activities of urban residents has the advantage of improving the accuracy of analysis and the utility of the results.

In Korea, the PM warning system has been implemented to reduce damage to the public by notifying the occurrence of high PM concentration, and guidelines for national action and measures are presented depending on the PM concentration. The central and local governments encourage individuals to stay indoors when there is a high PM concentration; however, it is impractical for individuals to refrain from going outdoors because of their work, essential activities such as those for livelihood, or recreational activities. Therefore, it is necessary to ascertain the effect of concerns over high PM concentrations on people’s participation in outdoor activities based on objective data. Furthermore, various policy approaches can be made by identifying regions where citizen activities have decreased due to high PM concentrations and regions where they have not. This can enable the central and local governments to concentrate their policy measures and efforts in regions where people’s activities have not decreased due to high PM concentrations.

Based on this background, this study spatially explores the effect of high PM concentration on the activities of urban residents, using high-resolution space–time population Big Data measured by recent telecommunication companies, and examines the effect of PM by statistically verifying it. First, this study examines the changes in the visiting population in Seoul during periods of high PM concentrations and analyzes the regional differences in these changes. Second, it examines the spatial characteristics that affect such regional differences (hotspots, cold spots) by conducting a spatial cluster analysis using GIS to examine the regional gap and logistic regression analysis to analyze the factors affecting regional differences.

## 2. Literature Review

The activities of urban residents interact with the built environment and constitute the vitality of the neighborhood [10]. Neighborhood vitality is an important factor for a livable and sustainable neighborhood [11,12]. Active urban residents’ activities are a source of vitality in the neighborhood, and urban vitality can be created when people move freely and communicate [13]. Such activities of urban residents also affect their physical health, and are affected by various factors such as the physical and social conditions of the neighborhood [14]. Many studies using quantitative data, such as [7,8,15], suggested that the activities of urban residents are affected by the physical environment of the neighborhood, such as land-use characteristics, roads and traffic, public transport accessibility, and infrastructure; however, empirical studies on the relationship between air pollution and environmental pollution are lacking.

The impact of air pollution varies depending on the period of exposure and activity type [16]. Various studies have proven that an increase in particulate matter (PM) and high PM concentrations reduces various urban activities such as outdoor activities, labor, and consumption. The authors of [4,17,18] discovered that individuals perceiving PM show negative emotions and physical symptoms, as well as avoidance behavior. According to [19], when perceived air pollution increased by one unit, 20% of physical activities decreased. Ref. [20] applied the theory of planned behavior by classifying PM risk perception into social, environmental, economic, and physical perception, and examined its effects on attitude, subjective norms, and perceived behavioral control. It was found that social and physical perception affected subjective norms, and thereby behavioral intention. The authors of [21] studied the relationship between PM concentrations and activity by examining changes in activities with respect to PM concentrations (μg/m^3^) by individual weight. They found that higher concentrations led to a greater possibility of reduced physical activity among people with normal weight and obesity. Moreover, Ref. [22] analyzed the effects of the Air Quality Index (AQI) and PM2.5 on activities and discovered that a 10-unit (μg/m^3^) increase in average PM2.5 concentrations at the regional level led to at least a 10% increase in the probability that there would be no activity. Similarly, Ref. [23] found that PM concentrations negatively affected the number of steps walked daily; however, the significance was not high. The authors of [24] collected data on the number of passengers accessing public transportation and air pollutant concentrations to analyze the effect of PM on human outdoor activities and predicted the decrease in the number of subway passengers with respect to increases in PM levels using machine learning. Additionally, Ref. [25] analyzed the relationship between passenger number and PM using the passenger data of each subway station in Seoul and proved that higher PM concentrations led to a reduction in the number of subway passengers.

The abovementioned studies proved that an increase in PM increases individual risk perception and anxiety, and thereby reduces physical or outdoor activities. While many studies have been conducted at the individual level, few have examined the population level or spatial differences. Since the impact of PM varies depending on characteristics (age, social and economic characteristics, etc.) at the individual level, it may also vary at the spatial level depending on the concentration of people’s activities, regional use, or features. Therefore, it is necessary to approach the impact of PM from multiple perspectives [16].

Recently, Big Data have been actively used in research on PM. Ref. [26] analyzed real-time PM exposure level using mobile Big Data, and in [27,28], the PM concentration and air pollutants were predicted using artificial neural networks. Ref. [29] constructed Big Data using portable air-quality-measurement equipment and mapped the air quality in high resolution. Ref. [30] reviewed studies that predicted air quality and suggested machine-learning methods such as Big Data, artificial intelligence, support vector machine, and random forest with high usability. Ref. [31] analyzed the effects of natural and socioeconomic factors on the PM concentration using remote sensing and geospatial Big Data. Ref. [32] analyzed people’s perception of PM using social Big Data and suggested risks. Using social media and satellite Big Data on the prevalence and mortality by disease, Refs. [32,33] revealed that PM increases the incidence and mortality of diseases. As such, many studies related to PM use Big Data, but they are focused on research that predicts air quality using machine learning, or analyze the relationship between the mortality rate with PM.

This study is differentiated from previous studies in the following ways: first, this study expands the contextual scope of previous studies that focused exclusively on individual activities using survey data. Thus, this study analyzes changes in the number of visitors as the aggregation of individual outdoor activities. It also spatially analyzes data regarding visitors to empirically ascertain the regions that demonstrate many changes and regions that do not. Additionally, the spatial characteristics that affect these regional differences are also examined. Undertaking such research was hindered in the past by difficulties in obtaining population data that can examine people’s activities. This study was enabled by recent developments in smart technology that utilizes sensor-based Big Data on the floating population; these Big Data are expected to have various implications for urban environment studies.

## 3. Materials and Methods

### 3.1. Research Sites and Research Scope

This study selected Seoul, a highly dense city (16,185 persons/km^2^) with a population of about 9.795 million people (approximately 19% of the entire South Korean population) as of 2021, as the research site [34]. Seoul was selected because labor, leisure, and consumption-related activities can be widely observed there; thus, it is possible to examine changes in the activities of urban residents with regard to PM. Additionally, data collection is facilitated because the city is equipped with sensor-based facilities to collect the floating population’s Big Data. The spatial scope of this study includes all areas of Seoul, and the research was conducted using “dong,” which is generally perceived as the neighborhood unit, as the unit of analysis. Seoul is comprised of 25 “gu” districts, each of which is comprised of multiple dongs. Overall, there are 424 dongs in Seoul, with an average of 23,048 people living in each dong.

The temporal scope of this study was 6 months between 1 November 2018 and 30 April 2019, since PM mostly occurs in fall, winter, and spring in Korea. Additionally, the pre-COVID-19 period is selected to ensure the generalizability of the results. The daily and hourly data on the visiting population in the city were collected and used in the analysis. The analysis was conducted by dividing the days into weekdays and weekends, as the activities of urban residents tended to vary depending on the day of the week—mandatory activities such as studying and learning predominantly took place on weekdays, while outdoor activities such as those for leisure took place on weekends.

### 3.2. Using Big Data of the de Facto Population to Identify Visitor Activity

Most studies examining changes in the activities of urban residents with PM used survey data. However, the emergence of Big Data that can check the hourly de facto population from mobile base stations due to recent innovations in information and communications technology (ICT) and sensors has enabled scholars to overcome the limitations of survey data on samples. The data used in this study were based on the de facto population measured every hour at the base stations of a Korean mobile carrier (SKT). The de facto population includes all individuals present within the area at a specified time and comprises of the resident, working, and visiting populations. This study used the visiting population as the variable representing the outdoor movement and outdoor activity of urban residents and excluded resident and working populations who mostly conduct indoor activities. The raw data were built by the hour, but 6:00 to 21:00 h was set as the activity time after considering the citizens’ living patterns, as this is when most residents carry out their daily routines. Based on the hourly visiting population data of that time zone, the average hourly visiting population for each day was calculated. These data divided Seoul into 200,515 50 × 50 m cells, derived through geocoding and GIS spatial operations, for the analysis. The visiting population was divided by area to calculate the average hourly visiting population density per dong, because of the difference in area per administrative dong. The average hourly visiting population density per administrative dong (person/km^2^) was used as the key data for spatial and statistical analysis models by calculating the difference between high concentration and control days.

### 3.3. Days with High PM Concentrations and Control Days

Particulate matter (PM) in the air with diameters of 10 μm or less and 2.5 μm or less are classified into PM10 and PM2.5, respectively. In Korea, PM warnings are issued when the PM concentration increases to levels harmful to public health. For PM_10_, a watch is issued when the average hourly concentration at the air-quality-monitoring station is 150 μg/m^3^ or higher for at least 2 h, and a warning is issued when the average hourly concentration is 300 μg/m^3^ for at least 2 h. For PM2.5, a watch is issued when the average hourly concentration is 75 μg/m^3^ or higher for at least 2 h, and a warning is issued when the average hourly concentration is 150 μg/m^3^ for at least 2 h [35]. This study selected the days on which the watch and warning for PM10 and PM2.5 were issued as the days with high PM concentrations.

This study first identified the days with high PM concentrations, since the key variable is the difference in the average hourly visiting population between days with high PM concentrations and control days. During the research period from November 2018 to April 2019, there were 35 days (24 weekdays, 11 weekends) with high PM concentrations, as shown in Table 1. While all days excluding the ones with high PM concentrations can be regarded as control days, this study excluded public holidays and days with fresh snow cover, as they are outliers in visit activity compared to the control days. The excluded days are presented in Table 1.

(1) $$HDVM_{i}=\frac{\sum HDV_{i}}{HDN}$$

*HDVM_i_* = Average visiting population density on high-concentration days in *i* administrative dong.

*HDV_i_* = Visiting population density on high-concentration days in *i* administrative dong.

*HDN* = Number of high concentration days.

(2) $$NDVM_{i}=\frac{\sum NDV_{i}}{NDN}$$

*NDVM_i_* = Average visiting population density on control days in *i* administrative dong.

*NDV_i_* = Visiting population density on control days in *i* administrative dong.

*NDN* = Number of control days.

### 3.4. Spatial Analysis and Logistic Regression Model

This study conducted a spatial cluster analysis using local indicators of spatial association (LISA) to spatially analyze the difference in the average hourly visiting population between days with high PM concentrations and control days. This methodology was developed by [36] and is used in various exploratory spatial data analyses (ESDAs). LISA gives weights between neighboring regions using Local Moran’s I statistic and calculates the intensity of the clusters based on the similarity of the weighted attribute values to identify similar cluster areas and distinct surrounding areas [37].

Clusters are classified into four types depending on the relationship between a specific region and its neighbors: H-H (High–High), H-L (High–Low), L-H (Low–High), and L-L (Low–Low). The H-H cluster represents a specific region and its neighbors that saw a significant increase in the density of the visiting population, despite having high PM concentrations, and is thus classified as a hotspot. The H-L cluster represents a condition where the specific exhibits show an increase in the density of the visiting population despite high PM concentrations, while the neighboring regions show a decrease. Conversely, the L-H cluster represents a situation where the central region shows a relatively greater decrease than its neighbors. The L-L cluster represents a situation where a specific region and its neighbors show a significant decrease in the visiting population, and thus, the region is classified as a cold spot.

This study used the binomial logistic regression model to analyze the spatial characteristics affecting the regions (dependent variables) that were hotspots or cold spots in the cluster analysis. The independent variables of the logistic regression model were land use [8,9,38,39], public transport accessibility [40,41], pedestrian environment [15,42,43], and living infrastructure [42,44,45]; these were selected based on previous studies that analyzed spatial characteristics affecting outdoor activities.

Land-use characteristics are variables to determine the primary use of a specific region and the ratio of the area used by residential, commercial, business and industrial facilities were selected as variables. Land-use mix was also included as a variable based on previous studies [46,47,48], which emphasized it as a key variable that increases the walking and outdoor activities of urban residents. Since public transport accessibility is a key factor affecting outdoor activities, this study included the number of bus stops and subway stations as variables. The length of the pedestrian path and Walk Score [49] were also included as variables of the pedestrian environment that have a significant effect on walking and outdoor activities. Walk Score measures the walkability of an environment and is calculated based on the accessibility of destinations such as cafes, libraries, and restaurants that induce walking, block length, and street connectivity. Finally, this study included the living infrastructure that affects people’s visit activity, such as park area ratio and the number of schools, hospitals, welfare facilities, markets, and public facilities per unit area.

The binomial logistic regression model measures the probability of an event’s occurrence depending on the conditions of the independent variables (X), by classifying the outcomes as maximum one or minimum zero in an S-curve logistic function. The odds ratio is used to interpret the logistic regression model, where the odds refer to the likelihood of a certain event occurring, and the odds ratio represents the ratio of odds that increase when the explanatory variable x increases by one unit. The details are provided in Table 2.

## 4. Results

### 4.1. Changes in Visiting Population during High PM Concentrations

The average hourly visiting population in Seoul was approximately 4804.94 per km^2^ on weekdays with high PM concentrations and 4871.65 per km^2^ on control days, thus showing a decrease of 66.71 due to high PM concentrations. A two-dependent-samples *t*-test was conducted to verify whether this change is statistically significant. The results in Table 3 indicate that there was a statistically significant difference in the visiting population between days of high PM concentrations and control days, proving that the visiting population decreases on days with PM. Since people mostly go to work or school on weekdays, with a focus on essential activities, they tend to reduce visits or outdoor activities other than essential activities when PM concentrations are high.

Visit activity was found to be higher on weekends than weekdays. The average hourly visiting population was approximately 5752.57 per km^2^, which is greater than the 5608.10 per km^2^ on control days, and showed a statistical significance on weekends with high PM concentrations (Table 4). This result contrasts with the hypothesis that high PM concentrations reduce visit activity, which can be interpreted in two ways. First, since people are actively engaged in various outdoor activities on the weekends despite high PM, which implies that outdoor activities on weekends are critical to livelihood, the central and local governments must take active measures to help people safely engage in outdoor activities instead of simple and passive measures such as urging restraint. Second, while weekends with high PM concentrations were mostly concentrated in January (when it was cold), there were almost no weekends with high PM concentrations in March and April (when it was relatively warm), implying that the increase in outdoor activities on warm spring weekends may have had an effect overall.

### 4.2. Spatial Pattern of Visiting Population Changes in High PM Concentrations

**Figure 1:**
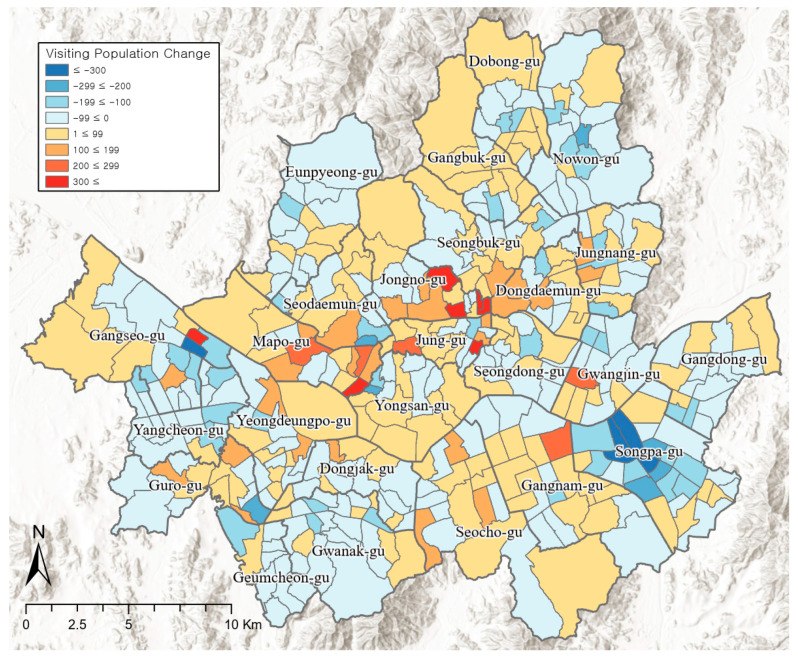
Spatial distribution of visiting population differences between weekdays with high PM concentrations and control days.

Figure 1 shows a heat map of the differences in the average hourly visiting population density (person/km^2^) between weekdays with high PM concentrations and control days in Seoul. Out of the 424 administrative dongs, 246 dongs showed a decrease in the visiting population, and the pattern was especially evident in Songpa-gu (Songpa 1-dong, Jamsil 3-dong, Jamsil 6-dong, Garak 1-dong), and Gangseo-gu (Gayang 3-dong). The visiting population showed a clear decrease in regions with concentrations of residential areas. However, despite the PM, 178 administrative dongs showed an increase in the visiting population, especially in the central parts of Seoul such as Jongno-gu (Jongno 5·6-ga-dong, Sungin 1-dong, Sungin 2-dong, Hyehwa-dong), and Jung-gu (Cheonggu-dong, Hoehyeon-dong). These regions have a low ratio of residential areas and a high concentration of business and commercial facilities. Previously, a review of the quantitative differences based on the density of the visiting population on weekdays proved that there was a statistically significant decrease; however, the actual spatial pattern shows that regions with decreases and increases appear simultaneously.

**Figure 2:**
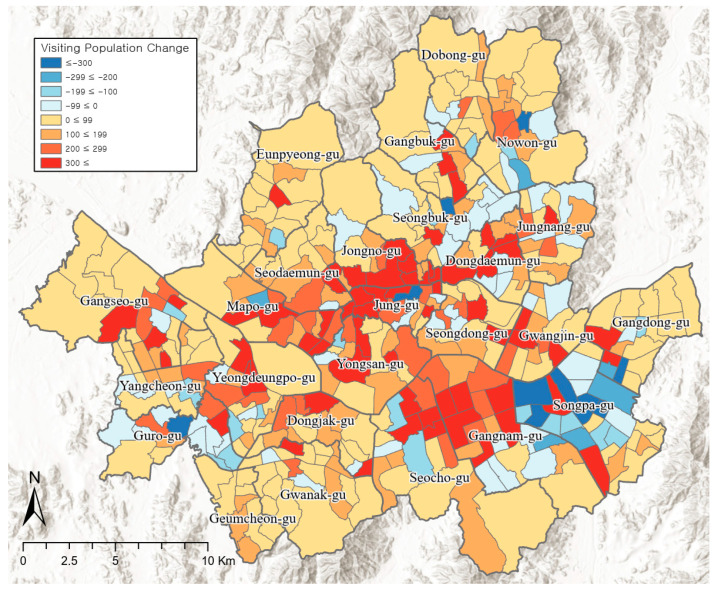
Spatial distribution of visiting population differences between weekends with high PM concentrations and control days.

The visiting population’s pattern on weekends with high PM concentrations and control days was different from that on weekdays. As shown in Figure 2, 333 administrative dongs showed an increase in the visiting population on high-concentration days, while 91 administrative dongs showed a decrease. Regions showing a clear increase in the visiting population were those including the central areas and sub-central areas such as Jongno-gu (Sungin 2-dong, Jongno 1·2·3·4·5·6-ga-dong), Jung-gu (Myeong-dong, Cheonggu-dong), and Mapo-gu (Seogyo-dong). In addition, the typical commercial and business area, Gangnam-gu, and the adjacent Seocho-gu also showed a significant increase in the visiting population. The region showing the greatest decrease in visiting population was Songpa-gu (Jamsil 6-dong, Songpa 1-dong, Bangi 1-dong, Jamsil 2-dong, Bangi 2-dong, Jamsilbon-dong). This could be because Songpa-gu is known to be a residential area for the middle or upper class in Seoul, and commercial and business facilities are concentrated in the surrounding Gangnam-gu and Seocho-gu regions.

LISA analysis was conducted to analyze the clustering patterns of the changes in the visiting population during high PM concentrations. The regions were classified into the H-H cluster (hotspots), where a specific region and its neighbors show an increase in the visiting population despite high PM concentrations, thus needing intensive management; the L-L cluster (cold spots) where a specific region and its neighbors show a clear decrease in the visiting population despite high PM concentrations; the H-L cluster, where a specific region shows an increase in visitors but its neighbors show a decrease; and the L-H cluster, where a specific region shows a decrease in visitors but its neighbors show an increase.

**Figure 3:**
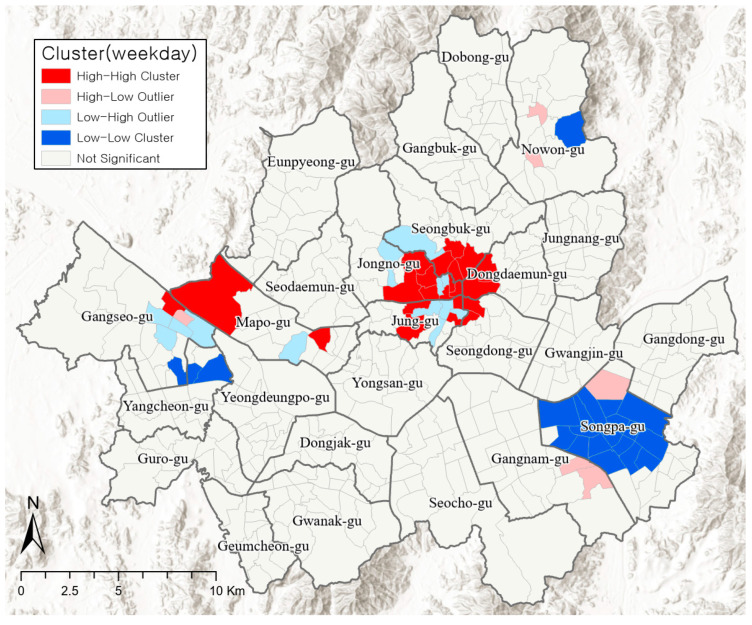
Clusters of visiting population changes on weekdays.

Figure 3 presents the results of the cluster analysis on changes in the visiting population on weekdays with high PM concentrations. There are two typical hotspots. The first is the cluster throughout Seoul’s main central area, Jung-gu (Euljiro-dong, Pil-dong, Jangchung-dong, Hwanghak-dong, Donghwa-dong) and Jongno-gu (Gahoe-dong, Jongno 1·2·3·4·5·6-ga-dong, Ihwa-dong, Changsin 1-dong, Sungin 1, 2-dong) and the neighboring areas of Seongbuk-gu, Dongdaemun-gu, and Seongdong-gu. The other is the cluster throughout Mapo-gu (Sangam-dong) and Gangseo-gu (Gayang 2-dong) in the west of Seoul. Cold spots where the visiting population clearly decreases on weekdays with high PM concentrations are mostly in Songpa-gu (Jamsil-dong, Seokchon-dong, Oryun-dong, Bangi-dong, Songpa-dong, Garak-dong, Samjeon-dong, Munjeong-dong), and partially in 1–2 dongs of Nowon-gu, Gangseo-gu, and Yangcheon-gu.

**Figure 4:**
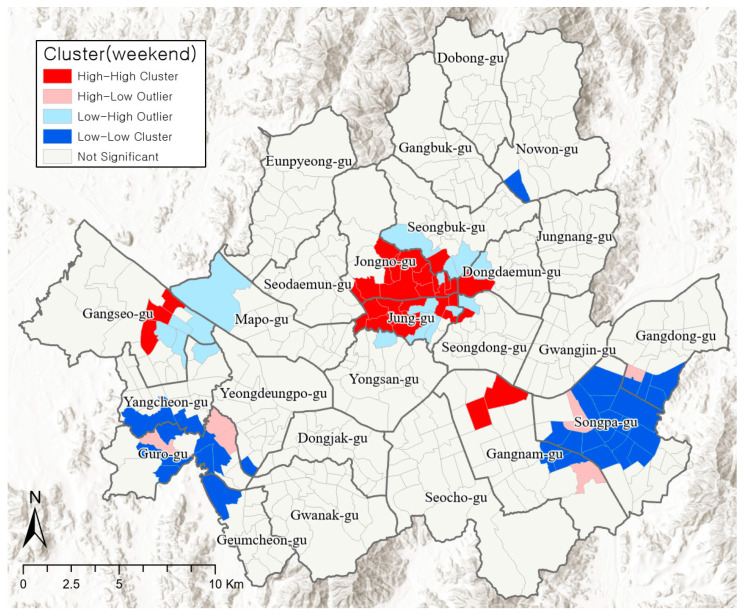
Clusters of visiting population changes on weekends.

Although the clusters of changes to the visiting population on weekends are similar to weekdays, there is a slight difference (see Figure 4). The hotspots are widely distributed throughout Jung-gu, Jongno-gu, Seongbuk-gu, Dongdaemun-gu, and Seongdong-gu. Cheongdam-dong, Samseong 2-dong, and Nonhyeon 2-dong of Gangnam-gu were observed to be clear hotspots on weekends. The 1–2 dongs of Gangseo-gu and Mapo-gu were partial hotspots. Cold spots or regions where the visiting population clearly decreases on weekends due to high PM concentrations were found in Songpa-gu, and some neighboring areas of Gangdong-gu and Gangnam-gu; thereby forming a bigger cluster. The transition patterns such as H-L and L-H were found in regions adjacent to hotspots and cold spots on both weekends and weekdays.

Jongno-gu and Jung-gu—hotspots that require policy interventions due to the clear increase in the visiting population despite high PM concentrations—are located at the very heart of Seoul, between the three urban centers of Seoul’s Master Plan [50]. Historical and cultural resources such as historical buildings, traditional streets, and hanoks (traditional houses) as well as commercial and business districts are concentrated in these regions; thus, there is high population mobility and activity in these regions from morning until the late hours. Gangnam, which is a hotspot on weekends, is also one of the three urban centers where commercial functions are highly concentrated. Furthermore, many young people gather in this region on weekends to access its commercial facilities and amenities.

Songpa-gu is a typical cold spot where people’s activities decrease the most on both weekdays and weekends. The residential area accounts for 62% of all areas, and it has the largest resident population (658,841) in a single gu. This region conforms to the characteristics of a residential area with a high resident population, owing to which outdoor activities reduce during periods of high PM concentrations.

The cluster analysis helped reveal that spatial characteristics such as land use and facilities affect visiting population changes during periods of high PM concentrations. The following section used a statistical analysis to empirically examine this observation.

### 4.3. Analysis of Factors Affecting Clusters Showing Visiting Population Changes

This study examined the effects of land use, public transport accessibility, pedestrian environment, and living infrastructure in hotspots (regional clusters where the visiting population increases despite high PM concentrations) and cold spots using binomial logistic regression analysis of the spatial clustering. The model’s validity was tested using the Hosmer–Lemeshow test, pseudo-R-squared test, predictive value classification, and receiver operating characteristic (ROC) curve before examining the results of the weekday and weekend logistics regression models. The Hosmer–Lemeshow test indicated that there was no statistically significant difference in the estimated probability and actual measured values of the models. The *p*-values of the weekday and weekend models were 0.159 and 0.887, respectively, thereby indicating that the estimated and measured values are similar. The pseudo-R-squared is verified by Cox–Snell R-squared and Nagelkerke R-squared. The result of Cox–Snell was 0.401 and Nagelkerke was 0.537 on weekdays, while on weekends, Cox–Snell was 0.424 and Nagelkerke was 0.569. The predictive value classification table shows the accuracy of the classification by comparing the predicted and measured values of the hotspots based on the model. Weekdays and weekends had a classification accuracy of 79.7% and 81.4%, respectively. Finally, the ROC curve derived the accuracy of the model using the area under the curve. Weekdays were 0.833 and weekends were 0.887, both of which were higher than 0.8, thus proving the model’s validity.

As indicated in Table 5, residential use, bus accessibility, length of pedestrian path, and the number of hospitals had a statistically significant effect as variables that affect hotspots. The results analyzed based on the odds ratio (Exp(B)) show that bus accessibility, length of pedestrian path, and the number of hospitals (with odds ratio greater than 1) are factors that increase the likelihood of a region being a hotspot. That is, regions with higher bus accessibility, better pedestrian paths, and more hospitals are likely to show an increase in the visiting population, even on weekdays with high PM concentrations. This could be attributed to how visiting the hospital is inevitable in many cases, although the use of other facilities in the living infrastructure tended to be optional. Conversely, the odds ratio of residential regions was smaller than one, indicating that it is a factor that increases the likelihood of a region being a cold spot. Regions with a high area ratio of residential use were more likely to exhibit a significant decrease in the visiting population on weekdays during periods of high PM.

The results of the logistic regression analysis on weekends are shown in Table 6. The residential use, commercial use, industrial use, bus accessibility, length of pedestrian path, and Walk-Score variables had a statistically significant effect on hotspots. The odds ratio (Exp(B)) shows that commercial use with an odds ratio greater than one is a factor that increases the likelihood of a specific region being a hotspot. This indicates that when the area ratio of commercial use increases by a unit, the probability that the region is a hotspot increases by 1.255 times. The odds ratio of residential and industrial use was smaller than one, indicating that they are factors that increase the odds that the region is a cold spot. Therefore, regions with high area ratios of residential and industrial use are likely to be cold spots, showing a clear decrease in the visiting population when there are high PM concentrations on weekends. Moreover, bus accessibility, length of the pedestrian path, and Walk Score all showed a statistically significant relationship with hotspots, indicating that weekend outdoor activities are concentrated in regions with high bus accessibility and a good pedestrian environment. Thus, people’s visits to these regions are likely to increase despite high PM concentration. Living infrastructures did not show a significant relationship with hotspots or cold spots on weekends.

## 5. Discussion and Conclusions

This study compared citizens’ outdoor activities on days with high PM concentrations and control days using Big Data of the de facto population collected from a mobile carrier and discussed the regional differences.

The results showed that changes in the number of visitors from high PM concentrations were different on weekdays and weekends. Weekdays showed an average decrease of 66.71 people per km^2^, indicating a statistically significant decrease, whereas weekends showed an increase in the visiting population despite high PM concentrations. This partially proves the hypothesis that PM reduces outdoor activities, thus proving that the effect of PM is not simple or comprehensive. The decrease was also observed to be spatially diverse. The results of the cluster analysis showed that Jung-gu and Jongno-gu—major urban centers of Seoul—are the hotspots exhibiting the clearest increase in the visiting population, despite high PM concentrations on both weekdays and weekends. These regions are Seoul’s key urban centers, with a high concentration of historical and cultural resources. Additionally, there is a high ratio of commercial districts with high population mobility and activity. Gangnam-gu is a hotspot on weekends and an urban center with high concentrations of commercial and business establishments. Additionally, it is a region where young people congregate on weekends. Conversely, Songpa-gu—with an extremely high ratio of residential use—was a cold spot, where the visiting population clearly decreased on both weekdays and weekends when there were high PM concentrations. This study also examined the spatial characteristics affecting hotspot and cold spot clusters using the binomial logistic regression analysis. Regions with more commercial use, higher bus accessibility, and better pedestrian environment (pedestrian path, Walk Score) were more likely to become hotspots, whereas those with higher ratios of residential use and industrial use were more likely to become cold spots. The number of hospitals among the living infrastructures also increased the probability of a region becoming a hotspot on weekdays.

The results have the following policy implications: first, the effects of high PM concentrations varied between weekdays and weekends and among regions. Modern society is actively engaged in leisure and outdoor activities on weekends; therefore, urging people to refrain from outdoor activities when there is PM is ineffective. More active efforts must be made to fundamentally reduce PM (such as managing the emission sources, supplying green transportation, installing roads and traffic facilities that prevent PM, etc.), install air-cleaning facilities to ensure that people conducting outdoor activities are not affected by PM, and intensively manage air quality in public facilities such as subways, railways, and airports. Second, since there are clear differences in the number of visitors across regions, it is necessary to establish customized measures by considering regional characteristics. Rather than applying a comprehensive policy across the country, measures must be undertaken in regions with a high ratio of commercial functions and a good pedestrian environment, as people prefer to participate in outdoor activities here. The policy must consider the land use, public-transport accessibility, pedestrian environment, and major facilities of the region since the findings have demonstrated their significance to visitation.

This study is significant as it empirically examined the effects of PM on the visit activity of urban residents, and the regional differences of these effects, a topic that had previously lacked empirical evidence. Further, it used Big Data based on communications services and demonstrated its feasibility as a source of data.

However, despite various efforts, this study has limitations. The temporal scope was limited to 6 months due to the high purchasing cost of Big Data. Moreover, this study was limited to spatial characteristics, although the outdoor activities of citizens are affected by factors such as weather and individual characteristics. Future research could add other variables and reflect on PM concentrations through direct analysis.

## Figures and Tables

**Table 1:** Days with high PM concentrations and days excluded due to outliers.

Classification		Weekdays	Weekends
Days with high PM concentrations (warning days)		November 6, November 7, November 27, November 28, December 21, January 14, January 15, January 23, February 7, February 22, February 25, February 28, March 4, March 5, March 6, March 7, March 12, March 20, March 21, March 27, March 28, April 5, April 22, April 23	December 22, December 23, January 5, January 12, January 13, January 19, January 20, February 23, February 24, March 2, March 3
Excluded	Days with fresh snow cover	December 13, February 1, February 15, February 19	November 24, December 16, February 16
	Public holidays	December 25, January 1, February 4–6, March 1	-

**Table 2:** Variables of the logistic regression model.

	Classification	Variable	Description	Source
Dependent variable		Hotspot = 1, Cold spot = 0	Hotspot: cluster of regions showing an increase in the visiting population when there are high PM concentrations Cold spot: cluster of regions showing a decrease in the visiting population when there are high PM concentrations	Derived from this study, based on the visiting population of the de facto population (SKT) data
Independent variable	Land use	Residential use	Area ratio of residential facilities	Building space information by use (Ministry of Land, Infrastructure, and Transport)
		Commercial use	Area ratio of commercial facilities	
		Business use	Area ratio of business facilities	
		Industrial use	Area ratio of industrial facilities	
		Land use mix	(Quasi-residential district +commercial district)/(Area of residential + commercial + industrial districts)	Land-use planning spatial data (Ministry of Land, Infrastructure, and Transport)
	Public transport accessibility	Bus accessibility	Number of bus stops per unit area	Seoul bus-stop location information (Seoul)
		Subway accessibility	Number of subway stations per unit area	Road-name address digital map (Ministry of the Interior and Safety)
	Pedestrian environment	Length of pedestrian path	Length of pedestrian path per unit area	Sidewalk/walkway (National Geographic Information Institute)
		Walk Score	Walkability index	[49]
	Living infrastructure	Park area ratio	(Park area/total area) × 100	Land-use zoning data/national land planning and spatial facilities/building space data (Ministry of Land, Infrastructure, and Transport)
		Schools	Number of facilities per unit area	
		Hospitals	Number of facilities per unit area	
		Welfare facilities	Number of facilities per unit area	
		Market	Number of facilities per unit area	
		Public facilities	Number of facilities per unit area	

**Table 3:** *t*-test results of visiting population changes on weekdays with high PM concentrations and control days.

Weekday	Average Hourly Visiting Population Density on Days with High PM Concentrations	Average Hourly Visiting Population Density on Days without High PM Concentrations
Mean	4804.94	4871.65
Standard deviation	3195.98	3203.82
N	424	424
t	−6.12	
*p* value	0.000	

**Table 4:** *t*-test results of visiting population changes on weekends with high PM concentrations and control days.

Weekend	Average Hourly Visiting Population Density on Days with High PM Concentrations	Average Hourly Visiting Population Density on Days without High PM Concentrations
Mean	5752.57	5608.10
Standard deviation	3851.90	3706.68
N	424	424
t	6.333	
*p* value	0.000	

**Table 5:** Results of logistic regression analysis (weekdays).

Variable		B	S.E.	Wald	Degree of Freedom	*p*-Value	Exp(B)
Land use	Residential use	−0.130	0.079	2.746	1	0.097 *	0.878
	Commercial use	0.138	0.179	0.593	1	0.441	1.148
	Business use	−0.415	0.518	0.642	1	0.423	0.660
	Industrial use	−0.538	1.051	0.262	1	0.609	0.584
	Land use mix	0.016	0.023	0.520	1	0.471	1.016
Public transport accessibility	Bus accessibility	0.063	0.036	3.146	1	0.076 *	1.065
	Subway accessibility	0.067	0.132	0.258	1	0.612	1.069
Pedestrian environment	Length of pedestrian path	0.001	0.000	4.084	1	0.043 **	1.001
	Walk Score	−0.057	0.058	0.990	1	0.320	0.944
Living infrastructure	Park area ratio	−0.025	0.024	1.119	1	0.290	0.975
	Number of schools	−0.039	0.048	0.643	1	0.423	0.962
	Number of hospitals	0.436	0.232	3.527	1	0.060 *	1.547
	Number of welfare facilities	−0.167	0.167	1.003	1	0.317	0.846
	Number of markets	0.124	0.141	0.776	1	0.378	1.132
	Number of public facilities	0.210	0.619	0.115	1	0.734	1.234
Constant term		4.073	3.368	1.463	1	0.226	58.747

**, * are 1% and 5% significance levels, respectively.

**Table 6:** Results of logistic regression analysis (weekends).

Variable		B	S.E.	Wald	Degree of Freedom	*p*-Value	Exp(B)
Land use	Residential use	−0.156	0.069	5.082	1	0.024 **	0.855
	Commercial use	0.227	0.176	1.659	1	0.098 *	1.255
	Ratio of business areas	0.174	0.373	0.217	1	0.641	1.190
	Industrial use	−5.301	2.577	4.232	1	0.040 **	0.055
	Land use mix	0.034	0.035	0.973	1	0.324	1.035
Public transport accessibility	Bus accessibility	0.054	0.033	2.738	1	0.098 *	1.056
	Subway accessibility	0.125	0.121	1.068	1	0.301	1.134
Pedestrian environment	Length of pedestrian path	0.003	0.000	5.234	1	0.022 **	1.002
	Walk Score	0.126	0.060	4.478	1	0.034 **	1.135
Living infrastructure	Park area ratio	0.028	0.027	1.108	1	0.292	1.029
	Number of schools	0.004	0.041	0.010	1	0.921	1.004
	Number of hospitals	−0.037	0.213	0.031	1	0.861	0.963
	Number of welfare facilities	−0.163	0.157	1.084	1	0.298	0.849
	Number of markets	0.035	0.152	0.054	1	0.817	1.036
	Number of public facilities	0.074	0.471	0.025	1	0.875	1.077
Constant term		−8.514	3.460	6.054	1	0.014	0.000

**, * are 1% and 5% significance levels, respectively.

## Data Availability

Data sharing not applicable.
